# Long-Term BMI Trajectories and Category Changes in Older Mexican Adults: A 20-Year Longitudinal Analysis

**DOI:** 10.3390/epidemiologia7020051

**Published:** 2026-04-07

**Authors:** Israel Rico-Alba, Horacio Marquez-Gonzalez, Jessie Nallely Zurita-Cruz

**Affiliations:** 1 Facultad de Contaduría y Administración, Universidad Nacional Autónoma de México, Mexico City 04510, Mexico; mdricoalba@yahoo.com.mx; 2 Epidemiological Research Unit, Hospital Infantil de México Federico Gómez, Ministry of Health (SSA), Mexico City 06720, Mexico; horaciohimfg@gmail.com; 3 Facultad de Medicina, Universidad Nacional Autónoma de Mexico, Hospital Infantil de Mexico Federico Gómez, Mexico City 06720, Mexico

**Keywords:** BMI category, obesity, overweight, aging, older adults, cross-sectional analysis, Mexico, ENASEM

## Abstract

Background/Objectives: Body mass index (BMI) trajectories and transitions across adulthood are dynamic processes influenced by aging and social- and health-related factors, yet long-term patterns in older adults from middle-income countries remain insufficiently characterized. The objective of this study was to characterize long-term BMI trajectories and transitions, and to identify sociodemographic and clinical factors associated with adverse BMI patterns among Mexican adults aged ≥50 years followed over 20 years. Methods: This study used data from the Mexican Health and Aging Study (ENASEM), a nationally representative longitudinal cohort. Participants aged ≥50 years with repeated BMI measurements across survey waves were included. BMI trajectories and transitions between BMI categories were described, and multinomial regression models were used to examine factors associated with upward transitions and unstable high-BMI patterns. Results: Distinct BMI trajectory patterns were identified over the 20-year follow-up. Participants in stable normal-weight trajectories were younger, more frequently female, and had higher educational attainment and income. In contrast, those with stable overweight/obesity or fluctuating–adverse BMI patterns had higher baseline BMI and a greater prevalence of diabetes, hypertension, and multimorbidity. In multivariable analyses, age contributed to trajectory differences; however, sex, socioeconomic factors, baseline BMI, and chronic conditions remained independently associated with adverse BMI patterns. Conclusions: BMI trajectories in later life are heterogeneous and reflect the combined influence of aging, socioeconomic conditions, and chronic disease burden. Identifying groups at risk of adverse BMI patterns may support the development of targeted interventions to reduce obesity-related health consequences in older adults.

## 1. Introduction

Obesity is among the leading public health problems worldwide, and its prevalence has steadily increased in recent decades [1]. This increase is associated with a greater burden of noncommunicable chronic diseases, including type 2 diabetes mellitus, hypertension, cardiovascular disease, stroke, and various types of cancer, which together represent one of the major causes of global morbidity and mortality [2]. Moreover, obesity has functional and psychosocial consequences that significantly affect individuals’ quality of life and impose considerable costs on health care systems [3].

Several longitudinal studies have demonstrated that variations in body weight strongly influence the clinical course of individuals with metabolic and cardiovascular diseases [4,5]. Weight reduction is associated with improvements in cardiovascular risk factors, whereas the transition from normal weight to obesity significantly increases the 10-year risk of cardiovascular disease [4]. Similarly, a cohort followed for more than 14 years revealed that weight fluctuations among patients with type 2 diabetes were associated with differential risks of coronary and cardiovascular disease, with moderate weight gain even showing a potentially protective effect in older adults and those treated with sulfonylureas [5].

On the other hand, findings from clinical trials indicate that weight changes do not always translate into clinical benefits. In the EXAMINE study, conducted among patients with type 2 diabetes and a recent acute coronary syndrome, weight loss greater than 5% was associated with increased all-cause and cardiovascular mortality. In contrast, similar weight gains were linked to a higher risk of hospitalization for heart failure. These findings suggest that weight variations in this context may reflect disease severity rather than a direct causal mechanism [6]. This phenomenon, described as the “obesity paradox,” indicates that both excessive weight and unintentional weight loss are associated with adverse outcomes in populations with chronic comorbidities [6].

The impact of weight fluctuations has also been documented in population-based and context-based settings. During the COVID-19 pandemic, population studies in England revealed that rapid weight gain was more frequent among women, younger adults, individuals with lower socioeconomic status, and those with mental health conditions, highlighting the interplay between social, psychological, and behavioral factors in nutritional changes [7,8]. Clinically, analyses of body mass index (BMI) trajectories before and after type 2 diabetes diagnosis in real-world cohorts revealed a pattern of progressive weight gain prior to diagnosis, followed by subsequent weight loss, attributed to both therapeutic interventions and behavioral motivation triggered by diagnosis [9].

Conversely, it has been reported that not only weight gain but also fluctuations in BMI category may have adverse effects on morbidity and mortality among adults, including older populations; however, findings across studies remain inconsistent [10,11]. These discrepancies may be explained by age-related differences, as evidence suggests that the association between weight gain and mortality tends to weaken with advancing age throughout adulthood; in contrast, the link between weight loss—particularly from midlife to late adulthood—becomes more pronounced [12].

While most available evidence focuses on populations with chronic diseases, there is a notable knowledge gap regarding weight trajectories among apparently healthy older adults. In this group, both weight gain and unintentional weight loss have been associated with frailty, functional decline, and increased mortality, even in the absence of diagnosed metabolic disorders [3]. Identifying the factors that influence fluctuations in BMI category among individuals without chronic comorbidities is essential for understanding the social and biological determinants of healthy aging [3].

In Mexico, long-term changes in BMI trajectories must also be interpreted within the context of a profound nutritional transition characterized by increased consumption of energy-dense, ultra-processed foods, high intake of sugar-sweetened beverages, and reduced dietary quality. These shifts have been widely documented and are strongly associated with rising obesity prevalence and cardiometabolic risk across the life course, including in older adults, even in the absence of diagnosed chronic disease [1,13,14].

In this context, the present study aimed to characterize long-term BMI trajectories and transitions, and to identify sociodemographic and clinical factors associated with adverse BMI patterns among Mexican adults aged ≥50 years followed over 20 years.

## 2. Materials and Methods

### 2.1. Study Design and Data Source

A secondary analysis was conducted using data from the Mexican Health and Aging Study (ENASEM), a nationally representative longitudinal survey of adults aged 50 years and older. The study was designed using a multistage, stratified, probabilistic sampling framework. The baseline cohort was established in 2001 and included individuals born in 1951 or earlier, as well as their spouses, regardless of age. To maintain representativeness of the population aged ≥50 years, new subsamples were incorporated in 2012 (participants born between 1952 and 1961) and in 2018 (participants born between 1962 and 1968) [15,16,17,18].

For the present analysis, data from the 2003, 2012, 2015, 2018, 2021, and 2024 waves were included. Participants for whom information was available for at least two of these assessments were considered. All interviews were conducted face-to-face by trained staff from the Instituto Nacional de Estadística y Geografía (INEGI). In cases where participants had died, data were collected through proxy informants. ENASEM is a publicly accessible database supported by the National Institute on Aging (NIA) and INEGI.

Adults aged ≥50 years with at least two valid body mass index (BMI) measurements during follow-up were included. Records without valid weight or height data, implausible BMI values, or zero sampling weights were excluded.

### 2.2. Study Variables

The dependent variable was BMI category, defined by BMI and categorized as follows: underweight (<18.5 kg/m^2^), normal weight (18.5–24.9 kg/m^2^), overweight (25.0–29.9 kg/m^2^), and obesity (≥30 kg/m^2^).

The independent variables included the following:

- Demographic characteristics: age (years), sex (male/female), marital status (married/union vs. other), and native speaker of an indigenous language (yes/no).
- Socioeconomic factors: educational attainment (≤9, ≥10 years), annual household income (reported in U.S. dollars, adjusted for the corresponding Mexican peso exchange rate; 1 U.S. dollar (USD) = $10.8 Mexican pesos), and health coverage (public, private, both, or none).
- Health conditions and multimorbidity: self-reported physician diagnoses of hypertension, diabetes, cancer, chronic respiratory disease, cardiovascular disease, stroke, arthritis, fractures, and depression. A multimorbidity index was constructed as the sum of reported conditions.
- Psychosocial factors: depressive symptoms.

### 2.3. Study Population

Among the 14,366 participants included in the 2001 baseline cohort, 6541 individuals (45.5%) remained in follow-up by 2021. For the longitudinal analysis of BMI category fluctuations, only participants with complete and valid anthropometric data across all evaluation waves were included.

A total of 4028 participants were excluded due to missing weight or height data, nonparticipation in one or more intermediate waves, inconsistent information, zero sampling weights, or physiologically implausible BMI values. Consequently, the final analytic sample consisted of 2513 participants with valid BMI measurements across the six waves (2001, 2003, 2012, 2015, 2018, and 2021). For the statistical analysis, 13 additional cases with incomplete covariate data were excluded, resulting in a final sample of 2500 participants. This subsample ensures longitudinal comparability for evaluating fluctuations in BMI category and associated factors over the 20-year follow-up period.

### 2.4. Comparative and Classification Procedures

Participants were classified according to weight fluctuation patterns across the six survey waves, forming six distinct groups:

1. Stable Normal Weight: Individuals who started with normal weight and remained within the normal range.
2. Stable Overweight/Obesity: Those who began with overweight or obesity and remained in the same category.
3. Weight Gain Group: Participants who started with underweight or normal weight and transitioned to overweight or obesity during follow-up.
4. Weight Loss Group: Those who began with obesity and ended with overweight or normal weight.
5. “Fluctuating–improving” individuals who began with overweight or obesity, changed categories in at least two waves, and ended in a lower BMI category than they started.
6. “Fluctuating–adverse” individuals whose BMI category changed in at least two waves and who were overweight or obese, regardless of their initial status.

For this analysis, participants classified as underweight were excluded due to the small sample size (*n* = 15), which could have introduced classification bias and reduced the reliability of the statistical analysis.

Based on these classifications, participants considered to be at increased risk were those whose BMI fluctuated but ended as overweight or obese, those who remained as overweight or obese throughout follow-up, and those who transitioned upward and were overweight or obese at the end of the study period.

### 2.5. Statistical Analysis

The distribution of quantitative variables was verified using the Kolmogorov–Smirnov test. Categorical variables were summarized as weighted frequencies and proportions, accounting for the survey’s complex design. The Rao–Scott adjusted χ^2^ test was used to assess changes in distribution across survey waves.

To identify factors associated with fluctuations in BMI category during follow-up, analytical models were developed based on classification approaches to modify nutritional risk.

A multivariable logistic regression model (based on nutritional risk patterns) and a multinomial logistic regression model (based on weight fluctuation patterns, using the stable normal weight group as the reference category) were applied. Nutritional risk patterns or weight fluctuation groups were considered the dependent variables. All models were adjusted for age, sex, educational attainment, number of comorbidities, and the presence of diabetes and hypertension.

A significance level of *p* < 0.05 was established. All analyses were performed using Stata version 14.0 (StataCorp, College Station, TX, USA), applying the survey weights provided by ENASEM.

### 2.6. Ethical Considerations

This study is a secondary analysis of data from the Mexican Health and Aging Study (ENASEM), which is a publicly available, anonymized database. According to Mexican regulations and institutional policies, studies based exclusively on the analysis of publicly accessible, de-identified secondary data are exempt from review and approval by an ethics committee.

Specifically, this exemption is consistent with the Reglamento de la Ley General de Salud en Materia de Investigación para la Salud (Mexico), which states that research involving publicly available data without identifiable information does not require ethics committee approval. As such, no formal exemption letter is issued by an ethics committee for this type of study.

## 3. Results

### 3.1. Subsection

#### 3.1.1. Baseline Characteristics of the Study Population

At baseline (*n* = 2500), most participants were younger than 59 years (78.3%), and females represented 54.2% of the sample. Regarding educational level, 83.4% reported secondary education or less, while 16.6% had tertiary education. Speaking an indigenous language at home was reported by 4.8% of participants. In terms of annual household income, the majority (74.8%) earned between USD 926 and 23,150. Most participants (83.4%) were married or living with a partner, and 73% reported having public health coverage (Table 1).

The distribution of BMI category showed that 28.4% of participants were of normal weight, 43.6% were overweight, 27.4% were obese, and only 0.6% were underweight (Table 1).

With respect to multimorbidity, hypertension was the most prevalent condition (33.2%), followed by arthritis (15.7%) and diabetes (9.8%). Depression was reported by 28.9% of participants. The most frequent comorbidity combinations were isolated diabetes (46.7% of those with comorbidities) and diabetes with hypertension. A total of 29% of participants reported no comorbidities, while 10.4% reported four or more chronic diseases (Table 1).

#### 3.1.2. Changes in BMI Category over Time

**Figure 1:**
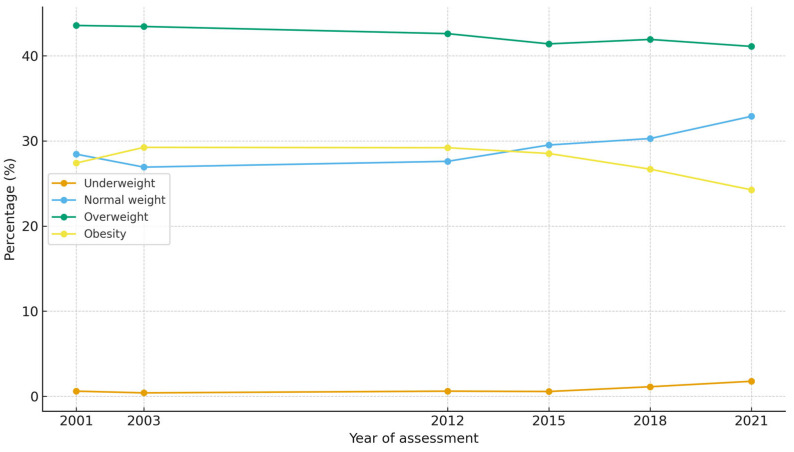
BMI category during follow-up (2001–2021).

Among the 2500 participants with complete BMI data across the six evaluation waves (2001, 2003, 2012, 2015, 2018, and 2021), relevant changes in BMI category were observed (Figure 1).

In the first interval (2001–2003), approximately two-thirds of participants (66.7%) remained in the same weight category, while 15.1% experienced a decrease and 18.2% an increase in nutritional classification. During the longer follow-up period (2003–2012), stability slightly decreased to 63%, although 18.9% of participants experienced a decrease in one category, and 18.0% showed weight gain.

In subsequent waves, a pattern of greater stability was identified. Between 2012–2015 and 2015–2018, approximately 70% of participants remained in the same BMI category, while the proportion who gained weight decreased to 13–14%, and reductions stabilized at approximately 16%. However, in the last follow-up interval (2018–2021), there was a renewed increase in downward transitions: 19.2% of participants lost weight, 67.2% remained stable, and 13.6% gained weight.

When specific BMI trajectories were examined, participants with normal weight at baseline exhibited the greatest relative stability—approximately two-thirds (≈64%) remained in this category throughout the follow-up period. Nevertheless, approximately 27% transitioned to overweight, and 4% progressed directly to obesity, suggesting a predominantly gradual, adjacent-category progression pattern.

Among those who were overweight at baseline, 61% remained in the same category, 23% regressed to normal weight, and 16% advanced to obesity, reflecting a bidirectional dynamic characterized by both recovery and deterioration. In contrast, obesity emerged as the most persistent state: approximately 72% of participants with obesity at baseline maintained this status, 24% shifted to overweight, and only 4% reverted to normal weight. Notably, the number of underweight individuals increased over time—from 15 at baseline to 44 at the final evaluation—indicating a net increase in this BMI category.

Temporal homogeneity analysis using χ^2^ tests revealed a biphasic population pattern in nutritional evolution across the 20-year period. No significant transitions were observed during the early intervals (2001–2003: χ^2^ = 3.54, *p* = 0.315; 2003–2012: χ^2^ = 1.42, *p* = 0.701; 2012–2015: χ^2^ = 2.28, *p* = 0.517; 2015–2018: χ^2^ = 6.52, *p* = 0.089), whereas a statistically significant change emerged in the final interval (2018–2021: χ^2^ = 9.37, *p* = 0.025).

Persistence analysis by nutritional category demonstrated that obesity maintained the highest statistical inertia, with probabilities of persistence of 0.74 in the initial period and 0.68 in the final period—which is consistent with its characterization as an absorbing state resistant to change. In contrast, normal weight showed increased stability over time (0.63 → 0.71), suggesting improved maintenance of this status in recent years. Overweight remained relatively constant (0.65 → 0.65), whereas underweight fluctuated slightly (0.27 → 0.36).

**Figure 2:**
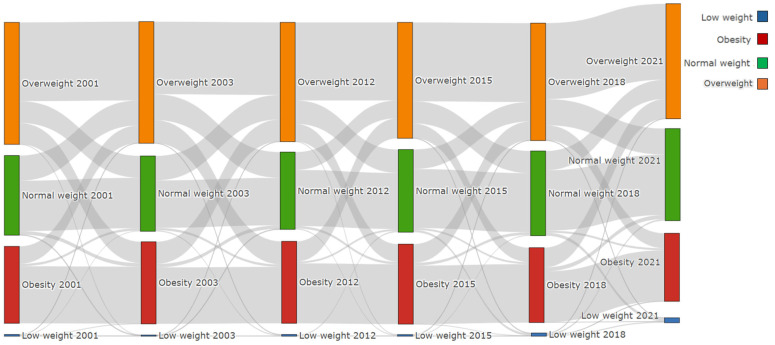
Transition of categories during follow-up (2001–2021).

Taken together, these longitudinal results indicate a net progression toward higher BMI categories over two decades, characterized by variable velocity—greater dynamism in the early years and relative stabilization thereafter—and increasing irreversibility of obesity once established. This pattern supports the notion that obesity is a chronic, self-sustaining condition with limited reversibility, that overweight is a transitional but unstable state, and that normal weight is the most stable nutritional trajectory across time (Figure 2).

Among participants who were underweight at baseline (*n* = 15), two remained underweight, nine achieved and maintained normal weight, and four progressed to overweight or obesity by the end of follow-up. With respect to fluctuation patterns, those who ended up in the overweight or obesity categories exhibited marked variations in BMI category throughout the 20-year follow-up period. In contrast, participants who reached and maintained a normal weight showed stable trajectories after improving their BMI category.

#### 3.1.3. Weight Fluctuation Patterns and Baseline Characteristics

Participants were classified into six distinct groups according to longitudinal weight fluctuation patterns across the six survey waves: (1) stable normal weight (*n* = 215, 8.6%), (2) stable overweight/obesity (*n* = 412, 16.5%), (3) weight gain (*n* = 242, 9.7%), (4) weight loss (*n* = 310, 12.4%), (5) fluctuating–improving (*n* = 590, 23.6%), and (6) fluctuating–adverse (*n* = 731, 29.2%). Fluctuations in body weight, reflected as changes in BMI category, occurred more frequently among individuals who experienced transitions between BMI categories.

Baseline characteristics differed significantly across groups (Table 2). In descriptive analyses, compared to all other groups, participants in the stable normal weight group were younger, predominantly female, and had higher educational attainment and income (*p* < 0.001 for all). Conversely, those in the stable overweight/obesity and fluctuating–adverse categories were older, had higher BMI, and a greater incidence of diabetes, hypertension, and multimorbidity (*p* < 0.001). The weight gain group showed intermediate values, reflecting a gradual progression from normal weight to overweight. In contrast, participants in the weight loss and fluctuating–improving groups tended to be older and present more chronic conditions at baseline, suggesting that weight reduction is linked to aging or disease.

No statistically significant differences were observed among the groups for indigenous language (*p* = 0.163), and chronic illness such as cancer (*p* = 0.179), respiratory disease (*p* = 0.902), heart disease (*p* = 0.286), stroke (*p* = 0.905), or arthritis (*p* = 0.276). These findings indicate that the burden of cardiometabolic conditions—particularly diabetes and hypertension—was the primary driver of heterogeneity between groups rather than other chronic morbidities.

Multinomial logistic regression analysis examining the associations between sociodemographic and clinical variables across the six weight fluctuation patterns revealed several significant relationships. High educational attainment (≥10 years) was positively associated with all patterns except for weight loss (OR range: 1.62–1.93; *p* < 0.05). Being married or in a union increased the likelihood of belonging to the stable overweight/obesity, weight gain, and fluctuating–adverse groups (OR = 2.40, 1.79, and 1.61, respectively; *p* < 0.05). Participants with a greater number of comorbidities were more likely to exhibit either weight gain (OR = 1.22; 95% CI: 1.02–1.45) or stable overweight/obesity patterns (OR = 1.15; 95% CI: 0.97–1.35). Older age was inversely associated with the stable overweight/obesity and weight gain groups (OR = 0.55 and 0.58, respectively; *p* < 0.05). Public health coverage was related to higher odds of belonging to the weight gain group (OR = 1.68; 95% CI: 1.15–2.44). Participants with coexisting diabetes and hypertension were significantly more likely to be classified in the weight loss group (OR = 4.41; 95% CI: 1.04–19.35), whereas those with diabetes alone had lower odds across most fluctuation patterns (*p* < 0.05) (Table 3).

The multivariable logistic regression model evaluating predictors of an adverse weight fluctuation profile indicated that being married or in a union (OR = 1.77; 95% CI: 1.59–1.94; *p* = 0.015), female sex (OR = 1.33; 95% CI: 1.11–1.77; *p* < 0.001), public health coverage (OR = 1.24; 95% CI: 1.04–1.46; *p* = 0.012), baseline overweight/obesity (OR = 1.78; 95% CI: 1.56–2.07; *p* < 0.001), diabetes (OR = 2.66; 95% CI: 2.00–3.55; *p* < 0.001), hypertension (OR = 2.03; 95% CI: 1.54–2.67; *p* < 0.001), and the coexistence of diabetes and obesity (OR = 1.56; 95% CI: 1.06–2.29; *p* = 0.015) were independently associated with an increased risk of adverse weight changes. The number of comorbidities also had a modest but statistically significant effect (OR = 1.14; 95% CI: 1.04–1.21; *p* = 0.001) (Table 4).

Taken together, the results demonstrate that adverse or persistent weight fluctuation patterns are more prevalent among individuals with preexisting metabolic risk factors, particularly diabetes and hypertension, whereas higher education and marital stability appear protective. The lack of association with other chronic diseases (cancer, respiratory, cardiovascular, or arthritis) supports the specificity of metabolic and sociodemographic determinants in driving long-term nutritional transitions. These findings suggest that female sex, normal weight at baseline, and a higher multimorbidity burden were the strongest factors associated with an unfavorable evolution of BMI category, underscoring the interaction between individual and clinical determinants in BMI trajectories over time.

## 4. Discussion

In this study, which was conducted over two decades of follow-up, most adults aged ≥50 years maintained stable BMI category, although relevant transitions were observed during specific periods, particularly between 2001–2003 and 2018–2021. These intervals showed the most significant changes, with an increase in the proportion of participants who lost weight during the last period and a higher rate of upward transitions during the early years of follow-up. The larger weight fluctuations observed during the two identified periods may reflect broader life-course and contextual changes occurring in adults aged 50 years and older rather than isolated individual behaviors. Over long follow-up intervals, individuals are likely to experience major transitions such as retirement, changes in household composition, declining income, and the onset or progression of chronic diseases, all of which have been associated with changes in body weight and weight stability in later life [19,20]. In addition, age-related alterations in body composition, including loss of muscle mass and redistribution of fat mass, may contribute to apparent weight instability even in the absence of substantial changes in total body weight [21]. Within the Mexican context, these individual-level processes occur alongside a well-documented nutritional and epidemiological transition characterized by increased availability of energy-dense, ultra-processed foods and reduced physical activity, which may further amplify long-term variability in BMI trajectories [13]. Importantly, these interpretations are exploratory and intended to contextualize the observed patterns rather than imply causal relationships.

Dietary quality represents an additional contextual factor that may contribute to the observed BMI trajectories. Although ENASEM does not include detailed dietary intake data, previous evidence indicates that poor diet quality—characterized by high consumption of ultra-processed foods and low intake of fiber-rich foods—is strongly associated with weight gain and obesity persistence in Mexican adults. In older populations, these dietary patterns may exacerbate metabolic vulnerability and limit the reversibility of obesity once established [13,14].

Obesity emerged as a persistent condition, with more than 70% of participants who began in this category remaining obese, while overweight individuals represented an intermediate, transitional state and were susceptible to both progression toward obesity and reversion to normal weight. These findings confirm the dynamic complexity of BMI trajectories in older adults, characterized by the coexistence of a stable subgroup and another with highly variable patterns, and underscore the importance of continuous longitudinal monitoring.

Although age differed significantly across BMI trajectory groups in descriptive analyses, multivariable models indicated that age alone did not explain the observed patterns. After adjustment, factors such as sex, educational level, income, baseline BMI, and the presence of chronic diseases remained independently associated with upward BMI transitions and unstable high-BMI trajectories. This suggests that weight trajectories in later life reflect the combined influence of aging and broader social and health-related factors rather than age alone.

Previous studies have shown that fluctuations in body weight among individuals with chronic diseases, such as type 2 diabetes mellitus or cardiovascular disease, are associated with adverse outcomes, including increased cardiovascular risk and mortality [5,6]. For instance, the Tehran Lipid and Glucose Study reported that weight changes in patients with type 2 diabetes had differential effects on the incidence of coronary heart disease [5]. In contrast, the EXAMINE trial revealed that both weight loss and weight gain were linked to adverse outcomes in patients with acute coronary syndrome [6]. Similarly, during the COVID-19 pandemic, rapid weight gain was observed among individuals with chronic comorbidities and social vulnerability factors [7,8]. However, unlike most previous research conducted in populations with preexisting chronic conditions, the present analysis was based on an open, community-based cohort of older Mexican adults, providing a broader and more representative view of weight dynamics in the general population.

Multivariate analysis revealed that female sex, baseline normal weight, and a greater number of comorbidities were the factors most strongly associated with the presence of a risk weight fluctuation pattern. These findings are partially consistent with previous studies showing that women tend to experience greater long-term weight gain trajectories and that starting at normal weight or overweight is associated with a latent risk of progression to obesity in the absence of preventive interventions [4,5]. Conversely, the association with multimorbidity reinforces the evidence that the coexistence of multiple chronic diseases not only reflects overall health burden but also contributes to greater vulnerability to unfavorable changes in BMI category [3].

An additional consideration in interpreting BMI trajectories in older adults is the progressive alteration in body composition that accompanies aging. Even in the absence of major weight changes, aging is associated with loss of skeletal muscle mass and bone mineral density, alongside increased fat mass and redistribution of adipose tissue, a condition often described as sarcopenic obesity [18,20]. These changes may result in stable or even declining BMI values while underlying metabolic risk persists or worsens. Importantly, regular physical activity and structured exercise interventions have been consistently shown to preserve lean mass, improve muscle strength, attenuate bone loss, and reduce fat mass in older adults, thereby improving body composition independently of substantial weight reduction [12,18,20]. Therefore, BMI changes alone may not fully capture the complexity of nutritional and metabolic transitions in later life [22].

The higher risk observed among women may be explained by specific physiological and social mechanisms. Hormonal fluctuations throughout the reproductive lifespan—including menstruation, pregnancy, lactation, and menopause—induce variations in appetite and energy metabolism, promoting greater fat accumulation in women than in men [18,23]. Moreover, decreased estrogen levels during certain life stages favor weight gain and alter fat distribution [23]. At a behavioral level, emotional eating—a strategy commonly used to cope with stress and a known risk factor for increased BMI and obesity—has been reported more frequently among women than among men [14,24]. This pattern may progress to binge eating, thereby combining dysregulated eating behavior with adverse metabolic consequences [16]. Additionally, women show higher levels of body image dissatisfaction and are more susceptible to sociocultural and social media influences that encourage both unhealthy restrictive diets and behaviors that perpetuate overeating [14,25,26,27,28]. Recent studies in Arab and Asian populations have confirmed that adult women more frequently engage in emotional eating and excessive intake in response to stress, supporting the idea that females represent a particularly vulnerable group for unfavorable nutritional trajectories [23,24].

Although overweight status has been shown to increase the risk of developing obesity and related chronic diseases over a 20-year follow-up period [29], maintaining a normal weight does not eliminate the possibility of progressing to obesity later in life. Lifestyle factors—particularly diet, physical activity, and adequate sleep—along with genetic predispositions, stress, and exposure to obesogenic environments, play a significant role in weight gain over time [22]. In this context, even individuals with normal weight may transition to overweight or obesity if unhealthy habits persist or physical activity remains insufficient. Among adults over 50 years of age, this risk is amplified by multimorbidity, which was identified in our study as an independent risk factor for adverse nutritional modification. Multimorbidity often limits functional capacity and reduces physical activity, promoting obesity progression. Collectively, these findings reinforce the multifactorial nature of obesity and highlight that maintaining a normal weight at one point in time does not guarantee future stability, which highlights the importance of adopting and sustaining healthy lifestyle behaviors throughout life.

The participants included in this study belong to a generation born in 1951 or earlier, whose childhood environment differed substantially from that of more recent cohorts [30]. During their early years, exposure to ultra-processed foods was limited, and the prevalence of childhood overweight and obesity was considerably lower than that in current generations. These contextual differences may partly explain the distinct patterns of BMI category fluctuations observed in this cohort compared with those reported among individuals born in the 2000s or later [30].

The present study has several limitations. First, this is a secondary analysis of an existing cohort; therefore, the variables were not originally collected with the specific objectives of this investigation in mind. Additionally, loss to follow-up may have introduced potential bias in the interpretation of the results. Other relevant factors, such as physical activity and overall dietary quality, were not assessed, which could have influenced the observed fluctuations in BMI category and should be considered when interpreting these findings. Moreover, detailed dietary intake parameters were not available in the ENASEM dataset, precluding a more precise evaluation of the relationship between specific nutritional patterns and BMI trajectories. Importantly, measures of body composition—such as lean mass, fat mass, or bone mineral density—were not available; therefore, we were unable to distinguish whether changes in BMI reflected alterations in adiposity, muscle mass, or bone mass. In addition, participants with cancer or other chronic conditions associated with unintentional weight loss were not excluded from the analysis. Consequently, some observed weight reductions may reflect disease-related or catabolic processes rather than intentional or health-related improvements, potentially affecting the interpretation of downward BMI transitions. Furthermore, the ENASEM cohort does not collect detailed information on participation in specific dietary interventions or the use of medications that may affect appetite or body weight; therefore, we were unable to account for the potential influence of weight-control diets or pharmacological treatments on BMI trajectories.

Despite these limitations, this study provides valuable longitudinal evidence on the dynamics of BMI category across adulthood and aging. The findings highlight the importance of early identification of individuals at risk for adverse nutritional fluctuations and reinforce the need for comprehensive strategies that integrate nutritional, metabolic, and lifestyle components to prevent long-term deterioration in health status.

From a public health perspective, our findings indicate the need to implement preventive strategies that focus on early detection and continuous monitoring of weight changes in older adults—even among those who initially have a normal weight. Identifying specific risk factors enables more effective and targeted interventions, particularly among women and individuals with multimorbidity, to reduce obesity progression and its associated complications. Importantly, these results suggest that interventions in older adults should not focus solely on weight reduction, but also on preserving functional capacity, muscle mass, and metabolic stability through integrated lifestyle and chronic disease management approaches. Strengthening lifestyle promotion programs and weight gain prevention strategies could help mitigate the impact of obesity as a persistent and difficult-to-reverse condition in this population. Moreover, further research is needed to analyze the patterns and determinants of changes in BMI category across different age groups, as our findings may not be generalizable to younger populations.

## 5. Conclusions

This 20-year longitudinal analysis revealed that BMI category in the aging Mexican population follows a dynamic yet increasingly irreversible trajectory, marked by early upward transitions in BMI and later stabilization of obesity as a chronic, self-sustaining condition. Normal weight was the most stable category. In contrast, overweight functioned as a transitional state with bidirectional movement—both improvement and deterioration—highlighting the fluid nature of intermediate BMI classifications over time.

The persistence of obesity across decades, with high inertia and limited reversibility, underscores its characterization as a metabolic “absorbing state” that consolidates health risks once established. In contrast, individuals who maintained a normal weight demonstrated higher educational attainment, younger age, and better socioeconomic conditions, suggesting a strong interplay between social determinants and nutritional resilience.

Adverse fluctuation patterns—defined by weight gain or unstable obesity trajectories—were most prevalent among older adults, women, and those living with diabetes, hypertension, or multimorbidity. These groups exhibited a significantly greater likelihood of remaining in or returning to elevated BMI categories, reflecting the synergistic effect of metabolic disease and aging on body weight regulation. Conversely, higher education and marital stability were protective factors against unfavorable trajectories, possibly reflecting greater health literacy, dietary control, and social support.

Overall, these findings highlight the dual nature of weight evolution across adulthood: while weight tends to be modifiable earlier in life, in later life stages, it is more difficult to overcome obesity and chronic disease. This biphasic pattern suggests that interventions should prioritize prevention and weight maintenance before the onset of metabolic comorbidities, as the probability of reversal diminishes once obesity is established. Understanding these long-term dynamics provides critical insight for designing age- and risk-tailored strategies to promote stable, healthy weight trajectories in middle-aged and older populations.

## Figures and Tables

**Table 1:** Description of the general characteristics of participants included at baseline according to BMI category based on BMI.

	Variables	All *n* = 2500	Underweight *n* = 15	Normal Weight *n* = 711	Overweight *n* = 1089	Obesity *n* = 685	*p*
Age group, *n* (%)							<0.001
	<59 years	1957 (78.3)	12 (80.0)	532 (74.8)	833 (76.5)	580 (84.7)	
	60–69 years	482 (19.3)	2 (13.3)	152 (21.4)	230 (21.1)	98 (14.3)	
	70–79 years	59 (2.3)	1 (6.7)	25 (3.5)	26 (2.4)	7 (1.0)	
	≥80 years	2 (0.1)	0 (0.0)	2 (0.3)	0 (0.0)	0 (0.0)	
Sex, *n* (%)							<0.001
	Female	1356 (54.2)	8 (53.3)	341 (47.9)	564 (51.8)	443 (64.7)	
	Male	1144 (45.8)	7 (46.7)	370 (52.1)	525 (48.2)	242 (35.3)	
Education, *n* (%)							<0.001
	≤9 years	2085 (83.4)	4 (26.7)	148 (20.8)	177 (16.2)	86 (12.5)	
	≥10 years	415 (16.6)	11 (73.3)	563 (79.2)	912 (83.8)	599 (87.5)	
Native speaker of an indigenous language, *n* (%)		119 (4.8)	2 (13.3)	39 (13.3)	49 (4.5)	29 (4.2)	0.222
Annual income (U.S. dollars), *n* (%)							0.219
	$0 to $925	475 (19.0)	5 (33.3)	137 (19.3)	212 (19.5)	121 (17.7)	
	$926 to $23,150	1871 (74.8)	9 (60.0)	523 (73.6)	814 (74.7)	525 (76.6)	
	$23,151 to $46,296	97 (3.9)	0 (0.0)	34 (4.8)	36 (3.3)	27 (3.9)	
	$46,297 to $69,445	25 (1.0)	1 (6.7)	6 (0.8)	10 (0.9)	8 (1.2)	
	>$69,446	32 (1.3)	0 (0.0)	11 (1.5)	17 (1.6)	4 (0.6)	
Marital status, *n* (%)							0.112
	Single/divorced/widowed	415 (16.1)	2 (13.3)	129 (18.1)	190 (17.5)	94 (13.7)	
	Married or in union	2085 (83.4)	12 (86.7)	582 (81.9)	899 (82.5)	591 (86.3)	
Health coverage, *n* (%)							0.015
	Public	1826 (73.0)	9 (60.0)	485 (68.2)	804 (73.8)	528 (77.1)	
	Private	21 (0.8)	0 (0.0)	208 (29.2)	261 (23.9)	149 (21.7)	
	Both	29 (1.2)	0 (0.0)	10 (1.4)	9 (0.8)	2 (0.3)	
	No insurance	624 (25.0)	6 (40.0)	8 (1.2)	15 (1.4)	6 (0.9)	
Multimorbidity, *n* (%)							
	Diabetes	246 (9.8)	0 (0.0)	56 (7.9)	95 (8.7)	95 (13.9)	0.001
	Cancer	42 (1.7)	1 (6.7)	12 (1.7)	12 (1.1)	17 (2.5)	0.068
	Respiratory disease	155 (6.2)	1 (6.7)	36 (5.1)	64 (5.9)	54 (7.9)	0.163
	Heart disease	63 (2.6)	0 (0.0)	20 (2.8)	23 (2.1)	20 (2.9)	0.611
	Stroke	26 (1.0)	0 (0.0)	7 (1.0)	12 (1.1)	7 (1.0)	0.994
	Arthritis	384 (15.7)	3 (20.0)	100 (14.1)	156 (14.3)	125 (18.2)	0.091
	Hypertension	829 (33.2)	2 (13.3)	172 (24.2)	347(31.9)	308 (44.9)	<0.001
Common patterns, *n* (%)							
	Diabetes & hypertension	129 (5.2)	0 (0.0)	20 (2.8)	45 (4.1)	64 (9.3)	<0.001
	Diabetes & obesity	151 (6.0)	0 (0.0)	56 (7.9)	95 (8.7)	0 (0.0)	<0.001
	Diabetes, hypertension & obesity	65 (2.6)	0 (0.0)	20 (2.8)	45 (4.1)	0 (0.0)	<0.001
Comorbid condition, *n* (%)							
	Depression	722 (28.9)	8 (53.3)	183 (25.7)	295 (27.1)	236 (34.4)	<0.001
Morbidities number, *n* (%)							<0.001
	0	725 (29.0)	7 (46.7)	262 (36.8)	319 (29.3)	137 (20.0)	
	1	682 (27.3)	4 (26.7)	204 (28.7)	316 (29.2)	158 (23.1)	
	2	509 (20.4)	1 (6.7)	138 (19.4)	219 (20.1)	151 (22.0)	
	3	325 (13.0)	1 (6.7)	65 (9.1)	137 (12.6)	122 (17.8)	
	≥4	259 (10.4)	2 (13.3)	42 (5.9)	98 (9.0)	117 (17.1)	

**Table 2:** Comparison of sociodemographic and clinical characteristics at baseline according to weight fluctuation trajectories during follow-up.

	Variables	Stable-NW *n* = 214	Stable-OW/OB *n* = 412	Gain *n* = 240	Loss *n* = 302	Fluct–Imp *n* = 589	Fluct–Adv *n* = 728	*p*
Age group, *n* (%) *								<0.001
	<59 years	164 (76.6)	358 (86.9)	206 (85.8)	208 (68.9)	432 (73.3)	577 (79.3)	
	60–69 years	41 (19.2)	51 (12.4)	31 (12.9)	81 (26.8)	132 (22.4)	144 (19.8)	
	70–79 years	8 (3.7)	3 (0.7)	3 (1.3)	13 (4.3)	24 (4.1)	7 (0.9)	
	≥80 years	1 (0.5)	0 (0.0)	0 (0.0)	0 (0.0)	1 (0.2)	0 (0.0)	
Sex, *n* (%) *								<0.001
	Female	98 (45.8)	263 (63.8)	147 (61.2)	151 (50.0)	285 (48.4)	404 (55.5)	
	Male	116 (54.2)	149 (36.2)	93 (38.8)	151 (50.0)	304 (51.6)	324 (44.5)	
Education, *n* (%) *								<0.001
	≤9 years	61 (28.5)	66 (16.0)	40 (16.7)	42 (14.9)	87 (14.8)	115 (15.8)	
	≥10 years	153 (71.5)	346 (84.0)	200 (83.3)	260 (86.1)	502 (85.2)	613 (84.2)	
Annual income (U.S. dollars), *n* (%) *								<0.001
	$0 to $925	42 (19.6)	69 (16.8)	31 (12.9)	80 (26.5)	112 (19.0)	136 (18.7)	
	$926 to $23,150	151 (70.6)	317 (76.9)	197 (82.1)	213 (70.5)	446 (75.8)	538 (73.9)	
	$23,151 to $46,296	16 (7.5)	18 (4.4)	6 (2.5)	3 (1.0)	17 (2.9)	37 (5.1)	
	$46,297 to $69,445	2 (0.9)	3 (0.7)	1 (0.4)	4 (1.3)	9 (1.5)	5 (0.7)	
	>$69,446	3 (1.4)	5 (1.2)	5 (2.1)	2 (0.7)	5 (0.8)	12 (1.6)	
BMI category, *n* (%) *								<0.001
	Normal weight	214 (100)	0 (0.0)	119 (49.5)	0 (0.0)	236 (40.1)	142 (19.5)	
	Overweight	0 (0.0)	178 (43.2)	121 (50.5)	224 (74.2)	114 (19.3)	452 (62.1)	
	Obesity	0 (0.0)	234 (56.8)	0 (0.0)	78 (25.8)	239 (40.6)	134 (18.4)	
Marital status, *n* (%) *								0.010
	Single/divorced/widowed	49 (22.9)	49 (11.9)	34 (14.2)	59 (19.5)	103 (17.5)	119 (16.4)	
	Married or in union	165 (77.1)	363 (88.1)	206 (85.8)	243 (80.5)	486 (82.5)	609 (83.6)	
Health coverage, *n* (%) *								<0.001
	Public	139 (64.9)	329 (79.8)	189 (78.7)	206 (68.2)	421 (71.5)	533 (73.2)	
	Private	2 (0.9)	3 (0.7)	5 (2.1)	2 (0.7)	4 (0.7)	5 (0.7)	
	Both	5 (2.4)	4 (1.0)	4 (1.7)	3 (1.0)	5 (0.8)	8 (1.1)	
	No insurance	68 (31.8)	76 (18.5)	42 (17.5)	91 (30.1)	159 (27.0)	182 (25.0)	
Multimorbidity, *n* (%)								
	Diabetes *	11 (5.1)	38 (9.2)	17 (7.1)	53 (17.5)	63 (10.7)	64 (8.8)	0.001
	Hypertension *	34 (15.9)	181 (43.9)	76 (31.7)	108 (35.8)	188 (31.9)	240 (32.9)	<0.001
Common patterns, *n* (%)								
	Diabetes & hypertension *	2 (0.9)	28 (6.8)	5 (2.1)	28 (9.3)	29 (4.9)	37 (5.1)	<0.001
	Diabetes & obesity *	11 (5.1)	12 (2.9)	17 (7.1)	33 (10.9)	29 (4.9)	49 (6.7)	<0.001
	Diabetes, hypertension & obesity *	2 (0.9)	5 (1.2)	5 (2.1)	16 (5.3)	11 (1.9)	26 (3.6)	<0.001
Morbidities number, *n* (%) *								<0.001
	0	97 (45.4)	88 (21.4)	73 (30.4)	73 (24.2)	170 (28.9)	217 (29.8)	
	1	59 (27.6)	104 (25.2)	59 (24.6)	90 (29.8)	171 (29.0)	195 (26.8)	
	2	36 (16.8)	89 (21.6)	56 (23.3)	57 (18.9)	131 (22.3)	139 (19.1)	
	3	11 (5.1)	68 (16.5)	29 (12.1)	52 (17.2)	69 (11.7)	95 (13.0)	
	≥4	11 (5.1)	63 (15.3)	23 (9.6)	30 (9.9)	48 (8.1)	82 (11.3)	

Stable-NW: Stable Normal Weight; Stable-OW/OB: Stable Overweight/Obesity; Gain: Weight Gain Group; Loss: Weight Loss Group; Fluct–Imp: Fluctuating–improving; Fluctuating–adverse: Fluct–Adv. * Statistically significant *p* values.

**Table 3:** Multinomial logistic regression examining factors associated with weight fluctuation patterns, with the stable normal weight group as reference.

Variables	Stable-OW/OB	Gain	Loss	Fluct–Imp	Fluct–Adv
Married or in union	2.40 * (1.48, 3.89)	1.79 * (1.07, 3.01)	1.59 (0.99, 2.56)	1.50 (0.98, 2.31)	1.61 * (1.06, 2.43)
Number of morbidities	1.15 (0.97, 1.35)	1.22 * (1.02, 1.45)	1.15 (0.97, 1.37)	0.98 (0.84, 1.15)	1.14 (0.98, 1.33)
Education ≥ 10 years	1.62 (1.05, 2.51)	1.91* (1.19, 3.08)	1.74 * (1.09, 2.77)	1.93 * (1.28, 2.89)	1.86 * (1.27, 2.73)
Age group	0.55 * (0.37, 0.81)	0.58 * (0.38, 0.89)	1.27 (0.90, 1.80)	1.19 (0.86, 1.63)	0.81 (0.59, 1.11)
Female sex	1.45 (0.99, 2.11)	1.48 (0.98, 2.23)	0.93 (0.63, 1.38)	0.94 (0.66, 1.34)	1.20 (0.85, 1.68)
Annual income	0.97 (0.72, 1.29)	1.03 (0.76, 1.40)	0.71 * (0.53, 0.98)	0.97 (0.74, 1.27)	1.02 (0.79, 1.32)
Health public coverage	1.38 (0.97, 1.97)	1.68 * (1.15, 2.44)	0.97 (0.67, 1.38)	−1.06 (0.77, 1.47)	1.14 (0.83, 1.55
Diabetes & hypertension	2.24 (0.51, 9.81)	1.12 (0.20, 6.08)	4.41 * (1.04, 19.35)	2.12 (0.48, 9.24)	2.54 (0.58, 10.96)
Diabetes	0.14 * (0.09, 0.22)	0.66 (0.41, 1.05)	0.33 * (0.21, 0.51)	0.19 * (0.12, 0.28)	0.42 * (0.28, 0.62)

* *p* < 0.05.

**Table 4:** Risk factors associated with the presence of risk weight fluctuation patterns.

		OR	95% CI	*p*
Variables				
	Married or in union *	1.77	1.59 to 1.94	0.015
	Number of morbidities *	1.14	1.04 to 1.21	0.001
	Age group	1.10	0.77 to 1.20	0.381
	Female sex *	1.33	1.11 to 1.77	<0.001
	Annual income	1.11	0.96 to 1.28	0.142
	Health public coverage *	1.24	1.04 to 1.46	0.012
	Overweight & obesity *	1.78	1.56 to 2.07	<0.001
	Diabetes *	2.66	2.00 to 3.55	<0.001
	Hypertension *	2.03	1.54 to 2.67	<0.001
	Diabetes & obesity *	1.56	1.06 to 2.29	0.015

* Statistically significant *p* values.

## Data Availability

The original data presented in this study are openly available in the Mexican Health and Aging Study (ENASEM) repository at https://www.inegi.org.mx/programas/enasem/ (accessed on 10 January 2025).

## Abbreviations

The following abbreviations are used in this manuscript:

ENASEM	Mexican Health and Aging Study
BMI	Body mass index
INEGI	Instituto Nacional de Estadística y Geografía
NIA	National Institute on Aging
Stable-NW	Stable normal weight
Stable-OW/OB	Stable overweight/obesity
Gain	Weight gain group
Loss	Weight loss group
Fluct–Imp	Fluctuating–improving
Fluct–Adv	Fluctuating–adverse
USD	U.S. dollar
